# Evaluation of the HIV drug resistance surveillance system in Mozambique, 2017-2018

**DOI:** 10.11604/pamj.2022.43.162.29206

**Published:** 2022-11-30

**Authors:** Samuel Nuvunga, Denise Chitsondzo Langa, Cynthia Semá Baltazar, Jahit Sacarlal, Erika Rossetto, Adolfo Vubil

**Affiliations:** 1 Field Epidemiology and Laboratory Training Program, National Institute of Health, Maputo, Mozambique,; 2 National Institute of Health, Maputo, Mozambique,; 3 Faculty of Medicine, Eduardo Mondlane University, Maputo, Mozambique,; 4 Mass Genics, Assigned to Mozambique Centers for Disease Control Prevention, Maputo, Mozambique

**Keywords:** Health information systems, antiviral drug resistance, HIV, surveillance evaluation, Mozambique

## Abstract

In the past ten years, the prevalence of primary Human Immunodeficiency Virus (HIV) drug resistance has ranged from zero to 25%, with higher and increasing rates in countries with access to antiretroviral therapy (ART), a specific case in Mozambique. World Health Organization (WHO) recommended that countries implement and routinely evaluate representative HIV drug resistance (HIVDR) research to monitor the emergency and transmission of HIV drug resistance mutations. This study aimed to describe the functioning of the system and also to identify gaps in the sensitivity, representativeness and quality of the data using the WHO methodology for Pre-Treatment and Acquired Approaches. We conducted a descriptive evaluation of the information system for surveillance of HIVDR in Mozambique in 2017-2018, based on updated guidelines for evaluating of public health surveillance systems from the Center for Disease Control and Prevention (CDC). The evaluation was conducted in all provinces using secondary data extracted from a cross-sectional survey database on HIVDR, with HIV positive cases at the beginning of ART aged ≥15 years. The system was described through informal conversations with HIVDR stakeholders and the simplicity, data quality and representativeness attributes were evaluated. With 322 positive cases at the beginning of ART (mean age=32.5 years, SD±11.1), about 63.0% (203/322) cases were women and 37.6% (121/322) men. The system was implemented in 25 health facilities distributed across all 11 Mozambican provinces and was considered representative. The system used two data collection instruments, the ART book and the form accompanying samples sent to the reference laboratory. The ART form, with 27 variables, was sent offline at two levels (health facility and National Institute of Health (NHI)), accompanied by dried blood spot samples for viral load testing and genotyping in the NHI virology laboratory, and was considered simple according to the standardized criteria. The system´s data quality was considered regular at 79.9%, with about 59.8% (1156/1932) of variable fields completed and 100% (1932/1932) consistency. The system used a single national laboratory to measure the prevalence of resistance to HIV drugs and was considered simple, with regular quality and representative data. We recommended public health efforts such as conducting genotyping tests be expanded to the provincial level, and periodic monitoring of system´s data collection procedures using forms.

## Introduction

The availability of antiretroviral therapy (ART) resulted in a reduction of morbidity and mortality associated with HIV/AIDS. However, the expansion of ART programs and long time of ART exposure in patients in treatment, has led to the emergence of mutation associated to HIV drug resistance [1,2]. The emergence of pre-Treatment and acquired drug resistance can negatively influence on viral suppression and consequently compromise the 90-90-90 global goals. In 2019, about 67% of people living with HIV worldwide received antiretroviral treatment, with 26% of people who started treatment infected with a virus resistant to first-line drugs (such as efavirenz) [3]. In the past ten years, the prevalence of primary HIV resistance has ranged from zero to 25%, with higher and increasing rates in countries with access to ART, a specific case in Mozambique [4]. The emergence of some resistance to HIV drugs (HIVDR) is inevitable in populations using ART, due to the error-prone nature of HIV replication [5]. The World Health Organization (WHO) recommends that countries routinely implement representative surveys of HIV drug resistance at the national level among different populations to monitor the emergence and transmission of drug resistance mutations [3].

The high prevalence of HIVDR in persons initiating ART, especially high prevalence of DRMs associated with resistance to NNRTIs, could require a change in standard first-line ART [6]. In addition, increase in the Cross-sectional Survey of HIV Drug Resistance. Mozambique's prevalence of pre-treatment drug resistance (PDR) over time may suggest programmatic deficiencies leading to undetected acquired HIVDR among persons on ART and subsequent increases in transmission of HIVDR to the uninfected population. The high prevalence of HIVDR in persons receiving ART, especially DRMs associated with resistance to NRTIs, could require a change in standard second-line ART [7]. In Mozambique, surveillance of HIV drug resistance has been carried out since 2007-2018, using sentinel post methodology in 4 health facilities located in the main metropolitan cities of Maputo and Beira, with pregnant women as the target population. As of 2017-2018, the country adopted the new WHO methodology, which advocates a research of HIV drug resistance mutations in a cross-sectional approach in pre-treatment population at a national level [8]. This study aimed to evaluate the information system for HIV drug resistance surveillance in Mozambique in 2017-2018 to determine its functioning and if it fulfills the objectives for which it was created, which was to inform the prevalence of resistance to HIV drugs in Mozambique.

## Program evaluation

### Methods

**Study design:** a descriptive evaluation of the information system for the surveillance of HIV drug resistance in Mozambique was carried out in the period 2017-2018, based on updated guidelines for the evaluation of public health surveillance systems at the Centers for Disease Control and Prevention [8]. A literature review was carried out by electronic consultation of works referring to the evaluation of the surveillance system in Brazil and the description of the system was made possible by reading the protocol of the cross-sectional research on drug resistance for HIV (HIVDR). The evaluation of attributes was based on surveillance data from the laboratory base of the National Institute of Health (INS), referring to HIVDR for the period 2017-2018. The recording instruments were also verified to count the existing variables and conversations with those involved in the cross-sectional survey of HIVDR surveillance to understand the functioning of the system.

**Data collection:** the assessment was carried out using the database of the Transversal Survey on HIV Drug Resistance (HIVDR) in Mozambique in the period 2017-2018 that integrates HIVDR surveillance. The description of the system took place through informal conversation with those involved in the information system for HIVDR surveillance to understand its operation and reading the protocol of the cross-sectional research on resistance to HIV drugs version 2.5 of December 21^st^, 2016 and informal conversations with those involved in the research.

**Definition of study variables:** the attributes simplicity, data quality and representativeness were evaluated. In simplicity, the number of variables on the form to be filled out, the form of data transmission, the levels of information transmission and the number of registration instruments to be filled out were observed. For the quality of the data, six variables out of the 27 existing in the database were randomly selected (age, sex, date of diagnosis, the quantity of CD4, starting date of ART and ART regimen) and observed to assess the completeness and consistency of data. Representativeness was assessed considering sex, age based on the National Institute of Statistics (NIS) and the number of health facilities that collect data for the study by provinces at the beginning of ART.

### Qualitative attributes

**Simplicity:** the simplicity of a public health surveillance system concerns its structure and ease of operation. Surveillance systems should be as simple as possible [9]. The simplicity of the survey information system for HIVDR surveillance was evaluated in terms of determining the number of variables on the form to be completed that detect a public health event and risk factors, based on the criteria, method of data transmission, method of filling in the data, levels of sending information and number of registration instruments to be filled out.

**Data quality:** data quality reflects the completeness and validity of data recorded in the public health surveillance system [9]. This attribute was evaluated based on the analysis of the percentage of records of mandatory and essential variables not filled in (completeness of data) and with incorrect filling in (data inconsistency). In this case, number of variables/fields filled in, completeness and coherence/consistency in filling in the data.

### Quantitative attributes

**Representativeness:** a representative public health surveillance system accurately describes the occurrence of a health event over time and its distribution in the population by place and person [9]. To assess this attribute, mandatory epidemiological variables were collected (period of data collection, location of data collection, target population of the survey distributed by sex and age groups) and number of sites in the country participating in the study.

**Statistical analysis:** descriptive analysis was performed using the SPSS version 24 statistical package, and figures and graphs executed using Microsoft Excel 2013 and QGIS version 3.10. For data analysis, descriptive statistics were performed, where frequency measures were calculated in the representativeness attribute to assess the proportion of national coverage of pre-ART cases, distribution of cases by sex and age groups. For the other variables, the proportion of cases verified to respond to the evaluation of each attribute was calculated, based on the defined criteria and on the parameters of each attribute. The measures of the evaluated attributes (simplicity, data quality and representativeness) are described in Table 1.

**Table 1:** evaluation criteria, parameters and results, Mozambique-2019

Attribute	Rating criteria	Parameter	Values achieved	Score
Simplicity	Determine the number of variables on the form to be filled out	≤ 27 variables - simple = 1 >27 variables - complex = 0	27 variables=1	0 - 5 points <3 complex ≥ 3 simple Achieved:5/5 Classification: Simple
	Data transmission method	On line - complex = 0 Off line - simple = 1	Offline=1	
	Method of filling in the data	Electronic - complex = 0 Manual - Simple = 1	Manual=1	
	Levels of sending information	≤ 2 - simple = 1 > 2 levels - complex = 0	2 levels = 1	
	Number of registration instruments to be filled	2 - simple = 1 > 2 - complex = 0	2 instruments = 1	
Data quality	% of fields filled (completeness)	High > 90% =1 Regular 70 - 89.0% =0.5 Low <70.0% =0	59.8%=0	0 - 2 points High = 2 Regular = 1 Low < 0 Achieved:1/2 Classification: Regular
	% Coherence / consistency in filling in data	High > 90% =1 Regular 70 - 89.0% =0.5 Low <70.0% =0	100%=1	
Representativeness	Distribution of cases by age, sex and provenance	Allows analysis by person, representative = 1 Allows analysis by place, representative = 1 Does not allow analysis by person and/or place, not representative = 0	Age and sex=1 Provenance=1	0 - 2 points: =2 representative < 2 = no representative Achieved:2/2 Classification: Representative

**Ethical considerations:** the study was implemented after approval by the National Institute of Health of Mozambique as of non-research activity. All data were anonymous and, as they were encoded secondary data, there was no risk of identification for the participants. Note that protocol version number 2.5 and informed consent were reviewed and approved by the National Bioethics Committee for Health of Mozambique, which is the National Institutional Review Board responsible for overseeing the study. Protocol violations and adverse incidents will be reported by an investigator from the National Bioethics Committee (CNBS) and CDC's Global AIDS Program in Mozambique and Atlanta.

### Results

**Figure 1:**
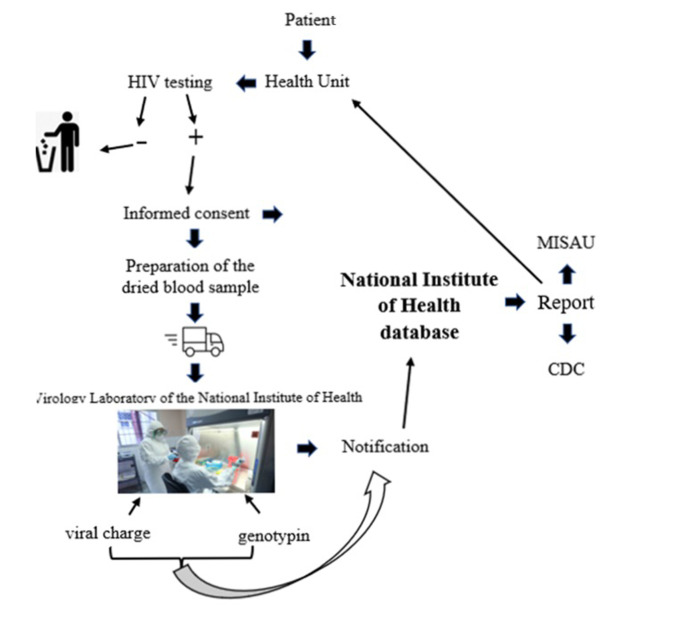
flowchart of data from the information system of the cross-sectional survey of HIVDR surveillance in Mozambique, 2019

**System description:** the information system for HIV drug resistance surveillance was designed in 2017-2018 from the Cross-Sectional Drug Resistance Survey for HIV a Generic Protocol was adapted for Mozambique, with the purpose of generating a representative estimate of the prevalence of drug resistance. The HIV Drug Resistance (HIVRD) surveillance information system started at the health unit with the collection of sociodemographic and clinical data using a form, followed by collecting the Dried Blood Sample (DBS) that accompanied the collected data and sent to the virology laboratory of the National Institute of Health (NIH). The data and samples collected at the level of health units in the provinces, regarding patients starting ART are sent to the central level (INS) and in turn are entered into the database and samples sent to the virology laboratory for viral load testing and genotyping. After the tests the results are entered into the same database (Figure 1).

***Evaluation of the attributes of the information system for HIVDR surveillance simplicity:*** this system used a form with 27 variables divided into three categories, namely: sociodemographic; clinical and laboratory exams, obtaining a maximum score of one point. The data was collected manually at the health unit level by trained personnel and were transmitted offline after collecting dried blood at the site. Then, the sample was packed together with the completed form and sent to the central level at the National Institute of Health's virology laboratory. This system worked with two data collection instruments, the TARV booklet in the health unit and the form that accompanies the samples to be sent to the reference laboratory. This attribute obtained a final score of 5/5, considering the simple system (Table 1).

***Data quality:*** for this attribute, six variables were chosen, namely age, sex, date of diagnosis, amount of CD4, date of starting ART and ART regimen. In 322 new cases of HIV at the beginning of ART reported in adults in the HIVDR database, it was observed for completeness that 59.7% (1156/1932) fields were complete and with 100% (1932/1932) of consistency. Regarding the TCD4 cell count, it was clear that of the 322, only 18.9% (61/322) had resulted in the database, where 13% (42/322) were < 350 cells/µL, 7.8% (25/322) were between 350-500 cells/µL and 12.7% (41/322)) were above 500 cells/µL (Table 2). This attribute obtained a general score of 79.9%, considering its data quality regular (Table 1).

**Table 2:** distribution of system data quality in Mozambique, 2019

variable	n= 1932 cells				
	existing cells	completeness	%	consistency	%
sex	322	302	93,8	322	100
age	322	303	94,1	322	100
diagnostic date	322	30	9,3	322	100
quantity of cd4	322	61	18.9	322	100
art start date	322	167	51,9	322	100
art regimen	322	291	90,4	322	100
overall	1932	1156	59,8	1932	100

**Figure 2:**
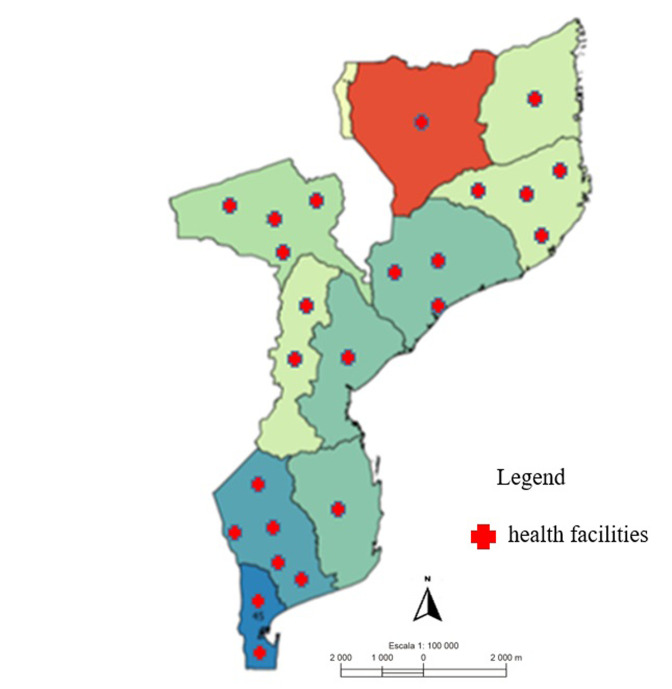
national distribution of hospitals involved in the information system for HIVDR surveillance in Mozambique, 2019

***Representativeness:*** the HIVDR surveillance system was represented in 25 health units distributed in 11 Mozambican provinces, namely: Niassa, Cabo Delgado, Sofala, Inhambane, Maputo Province and Maputo City with one health unit; Manica with two health units; Zambezia with three health units; Nampula and Tete with four health units and Gaza with five health units (Figure 2). Of the 322 HIV+ cases reported at the beginning of ART, with average age = 32.5 years and SD = ± 11.1, where 63.0% (203/322) were women and 37.6% (121/322) men, 24.8% (80/322) were 15-24 years old, 40.4% (130/322) 25-34 years old, 19.9% (64/322) 35-44, 9.9% (32/322) 45-54, 3.4% (11/322) 55-64 and 1.6% (5/322) were ≥ 65 years old. This attribute obtained a final score of two points, classifying the system as representative (Table 1).

### Discussion

We believe that our study is the first to evaluate the surveillance system for resistance to ART in Mozambique to provide support for the improvement of this surveillance, detecting possible gaps that could make it fragile with a view to improvement. This nationally representative study provided an overview of the quality of the HIVDR surveillance information system using the CDC/USA assessment method. The evaluation findings pointed out that the information system for HIVDR surveillance was considered simple with multidisciplinary involvement, from medical assistance to the suspicion of the case, adequate clinical management, and laboratory tests collection. However, in the study by Souza, 2010, divergent results were found where the surveillance system was considered complex - Brazil, 2007 [10]. The study emphasized that data transmission was designed to track Dried Blood Samples (DBS) for CD4 counting purposes as one of the risk markers of HIV infection progression [11]. Clinical and laboratory data were collected in two stages. In the first stage that occurs in health units, demographic data, CD4 and DBS count at central level (INS) are made available through accredited carriers, subjecting them to problems of delay and risk of loss during the transfer. In the second stage, which occurs at the NIH level, viral load and genotyping are performed, since the country has 8 laboratories with the capacity to carry out viral load [12], there is a need to reduce the workload at this level, leaving the task of measuring viral load at the provincial level during CD4 counting. Of 22.3% of the variables analyzed for the quality of data in the System, the percentage of fields filled in was low, these variables being essential for the production of information for triggering decision making.

The incompleteness of data from a surveillance system can compromise the monitoring of the event under surveillance, impairing the achievement of the objectives outlined by the program [12]. It is possible that the percentage of empty fields (incompleteness) may have been influenced by the volume of data handled by the data managers, since the data management was implemented only at the central level, with the need to adapt the System to the online model and the DBS sent separately with the identification code of each individual. The System reported a good representativeness which reflects an abrupt monitoring of time, person and place at country level, which allows to inform the situation of the event in surveillance, this study corroborates with the one carried out in Brazil with the similar finding [13]. It was reported in the representativeness of the system that the female sex presented a greater vulnerability to males, which can be linked to several factors, among them the cultural ones [14,15]. The age group from 25 to 34 years old presented the highest records in both sexes, which could indicate that this age group had the greatest vulnerability to HIV, however divergent studies with these findings showed that the group with the greatest vulnerability was between 15 and 24 years old (adolescents and youth) for both sexes [15,16]. Possibly, this difference can be justified by the demand for health services by medical care in the adult age group. In the result of this evaluation, some limitations related to the use of secondary data should be considered, such as the impossibility of evaluating the attribute positive predictive value (PPV) due to insufficient data and evaluation, the database did not include pregnant women, and final genotyping results. Although this evaluation has its limitations, it has contributed to boosting strategies to improve this surveillance system in Mozambique by identifying gaps in its functioning.

### Conclusion

This HIV surveillance allows the identification of cases of resistance to ART for decision making due to its operational ease, shows employee engagement, however, it raises the need to reinforce monitoring for the execution of activities in a timely and accurate way and has clear view of the resistance situation in all countries. There is a need to increase monitoring in order to guarantee a good quality of data, which could allow better design of national ART guidelines in Mozambique as the case for identifying the need for a change in standard first-line ART.
